# Preventive effect of aripiprazole once monthly on rehospitalization for bipolar disorder: A multicenter 1‐year retrospective mirror image study

**DOI:** 10.1002/npr2.12371

**Published:** 2023-08-10

**Authors:** Junichi Goto, Masahiro Shono, Yasuhisa Abe, Yasutaka Fujita, Keishi Ueda, Binchi Yoshida, Yoshihiro Nabeshima

**Affiliations:** ^1^ Yuge Neuropsychiatric Hospital Kumamoto Japan; ^2^ Yatsushiro Kosei Hospital Kumamoto Japan; ^3^ Kusatsu Hospital Hiroshima Japan; ^4^ Kumamoto Seimei Hospital Kumamoto Japan; ^5^ Hitoyoshi Mental Hospital Kumamoto Japan

**Keywords:** antipsychotic agents, bipolar disorder, delayed‐action preparations, patient readmission, retrospective studies

## Abstract

**Aim:**

We conducted a 1‐year retrospective mirror‐image study to investigate the effect of aripiprazole once monthly (AOM) on rehospitalization for bipolar disorder.

**Methods:**

Participants were recruited from psychiatric emergency and acute care hospitals in western Japan. We included 39 participants with bipolar disorder who had been administered AOM for at least 1 year with no missing medical records during the observational period. The primary outcomes were rehospitalization rate, number of rehospitalizations, total hospitalization days, and time to rehospitalization in the context of overall psychiatric readmissions. The significance level was set at *p* < 0.05.

**Results:**

AOM significantly reduced the rehospitalization rate from 23/39 (59%) to 7/39 (18%) (*p* = 0.001). The number of rehospitalizations decreased significantly from a mean of 0.85 per person‐year to 0.41 per person‐year (*p* = 0.048). The total hospitalization days significantly decreased from a mean of 34.9 days to 14.4 days (*p* = 0.008). AOM significantly prolonged the time to rehospitalization (*p* < 0.001).

**Conclusion:**

This study found that AOM reduces overall psychiatric rehospitalization for bipolar disorder based on data from 1 year before and after AOM administration in the real‐world setting. Future studies should examine the robustness and persistence of the rehospitalization preventive effect of AOM with larger sample sizes and longer observation periods beyond 1 year.

## Abstract 2

We conducted a 1‐year retrospective mirror‐image study to investigate the effect of aripiprazole once monthly (AOM) on rehospitalization for bipolar disorder. Our study found that AOM significantly reduced rehospitalization rates, number of rehospitalizations, and total hospitalization days and significantly prolonged the time to rehospitalization in the context of overall psychiatric readmissions among patients with bipolar disorder in the real‐world setting.

## Graphical abstract

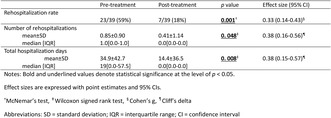

## 1 INTRODUCTION

Bipolar disorder is a prevalent mental health disorder, with an estimated frequency of approximately 2% worldwide.^1^ It is a chronic illness with recurrent episodes of manic, depressive, or mixed episodes throughout a lifetime.^2^ With each recurrence, the interval between them shortens, the disease stage progresses,^3^ and abnormalities in the neural network accumulate and lead to cognitive and psychosocial impairment—the so‐called neuroprogression.^4^, ^5^ Patients with bipolar disorder have lower work productivity and health‐related quality of life.^6^ Hence, bipolar disorder ranks 28th in years lived with disability and 67th in disability‐adjusted life years.^7^ Prevention of recurrence is clinically essential to avoid poor outcomes in patients with bipolar disorder.

Maintenance treatment with medication is crucial for preventing the recurrence of bipolar disorder. Treatment guidelines in various countries recommend maintenance treatment with mood stabilizers and antipsychotics.^8^, ^9^, ^10^ However, approximately 50% of patients with bipolar disorder have poor adherence.^11^ In a prospective 21‐month observational study in the treatment of bipolar disorder, the non‐adherent group had more recurrences, rehospitalizations, and suicide attempts than those of the adherent group.^12^ Using long‐acting injectable (LAI) other than oral medication improves medication adherence in bipolar disorder, as well as therapeutic alliances, shared decision‐making, and psychoeducation.^11^ A database study showed that the proportion of days covered (PDC) increased by 5% and the medication discontinuation rate decreased by 19% in LAI‐initiated patients with bipolar disorder compared with those in oral antipsychotic‐initiated patients with bipolar disorder.^13^

Once‐monthly long‐acting injection of aripiprazole, aripiprazole once monthly (AOM) has recently been approved of maintenance treatment of bipolar disorder. AOM prolonged the time to recurrence of any mood episode in a 52‐week, double‐blinded, randomized controlled trial of patients with bipolar disorder type I in the maintenance phase (hazard ratio: 0.45).^14^ Another 52‐week, open‐label study of AOM investigated patient‐oriented outcomes, and more than 70% of patients with bipolar disorder reported being extremely or very satisfied with AOM treatment, while more than 65% reported either no side effect or much less side effects compared with previous medications.^15^ Owing to its effectiveness and associated patient satisfaction, AOM has been recommended as a maintenance treatment for bipolar disorder by several pharmacotherapeutic guidelines.^9^, ^16^

Previous studies have shown that LAIs effectively reduce rehospitalizations for bipolar disorder in the real‐world setting. In a large Finnish cohort study, compared with identical oral antipsychotics, first‐generation antipsychotic LAIs and second‐generation antipsychotic LAIs (SGA‐LAIs) had an adjusted hazard ratio of 0.70 for psychiatric rehospitalization on bipolar disorder.^17^ One‐year, retrospective, mirror‐image studies have reported that both risperidone LAI and paliperidone LAI reduce rehospitalization in patients with bipolar disorder after their use.^18^, ^19^ Moreover, 1‐year, retrospective, mirror‐image studies conducted in an overall population of patients with bipolar disorder using three SGA‐LAIs—risperidone LAI, paliperidone LAI, and AOM—showed a statistically significant decrease in rehospitalization rates,^20^ the number of rehospitalizations,^21^ and total hospitalization days post‐treatment.^20^, ^21^, ^22^

However, previous studies have not verified whether AOM reduces rehospitalization for bipolar disorder after its use. A large Finnish cohort study included only risperidone LAI and olanzapine LAI but not AOM among SGA‐LAIs.^17^ In addition, 1‐year, retrospective, mirror image studies conducted in Turkey and Italy included a mixed group of patients with bipolar disorder using multiple SGA‐LAIs, such as risperidone LAI, paliperidone LAI, and AOM.^20^, ^21^, ^22^ These studies did not examine the effect of AOM alone on rehospitalization for bipolar disorder by selecting only AOM users.^20^, ^21^, ^22^, ^23^

Here, we conducted a 1‐year, retrospective, mirror‐image study to determine whether AOM reduces rehospitalization rates, number of rehospitalizations, and total hospitalization days and prolongs the time to rehospitalization in patients with bipolar disorder after its use. Our study examined the clinical impact of AOM by evaluating various types of readmissions, including overall psychiatric readmissions, readmissions specifically related to manic or depressive episodes, and both voluntary and involuntary readmissions. By analyzing these specific aspects, we aimed to provide a comprehensive understanding of the clinical benefits and effectiveness of AOM.

## 2 METHODS

### 2.1 Study design

**FIGURE 1:**
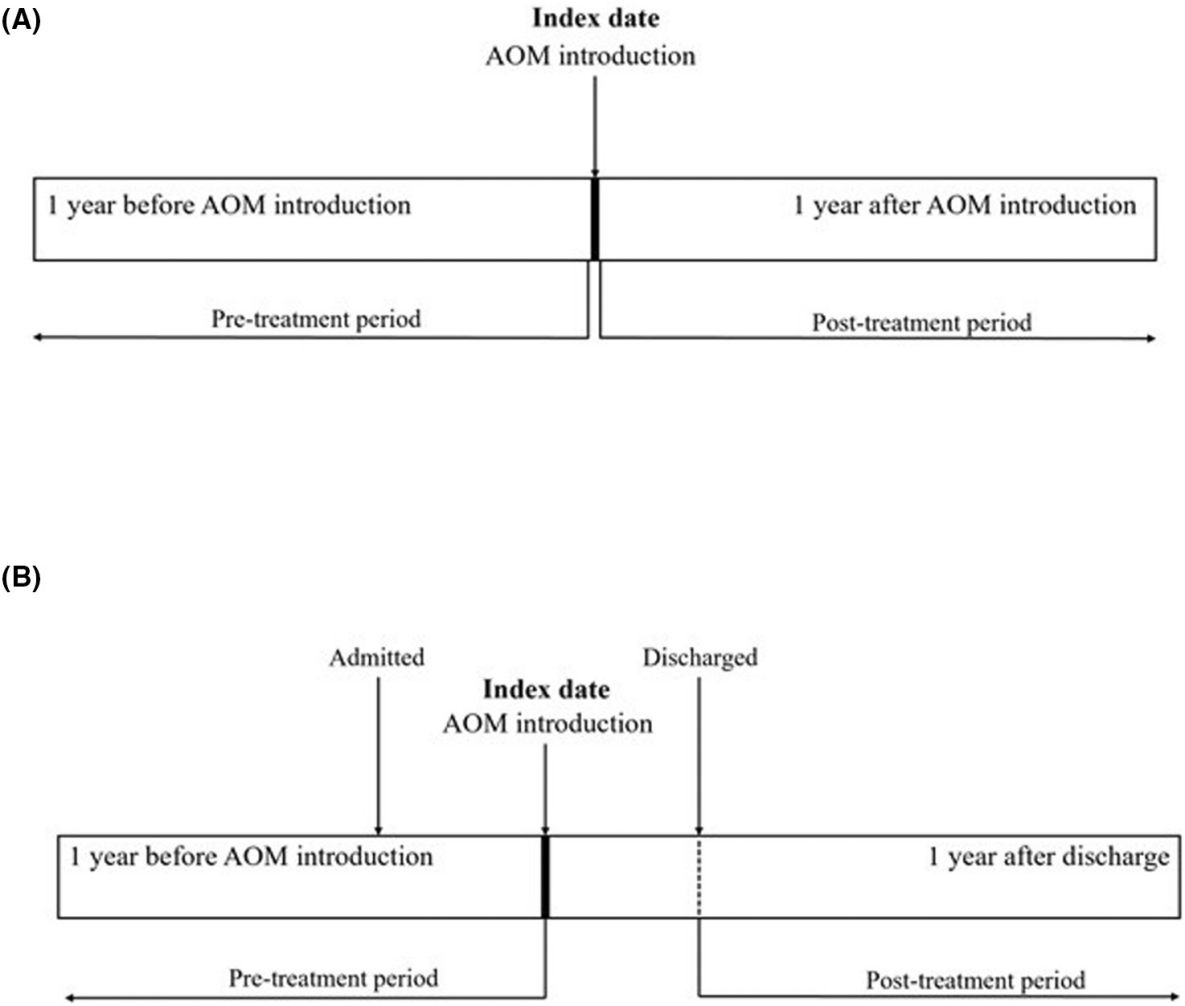
Definitions of observation periods for the mirror‐image study. (A) Observation periods for aripiprazole once monthly introduction in the outpatient setting; (B) Observation periods for aripiprazole once monthly introduction in the inpatient setting.

This 1‐year, multicenter, retrospective, mirror‐image study adhered to the Strengthening the Reporting of Observational Studies in Epidemiology guidelines.^24^ The study examined whether AOM introduction reduces rehospitalization in patients with bipolar disorder. Figure 1 shows the observation period of the study. The index date was the date of the first AOM administration. If AOM was introduced in an outpatient setting, the years before and after the index date were defined as the pre‐ and post‐treatment periods, respectively (Figure 1A). If AOM was introduced in an inpatient setting, the period up to 1 year before the index date was defined as the pre‐treatment period, whereas the year after discharge from the hospitalization in which AOM was introduced was defined as the post‐treatment period (Figure 1B); this was done because rehospitalizations cannot occur if patients have not first been discharged.

### 2.2 Setting of the study

The survey period was from January 1, 2023 to March 31, 2023. Six psychiatric emergency and acute care hospitals in Western Japan participated in this study. The information required for the study was retrospectively collected from the medical records of the participating hospitals.

### 2.3 Eligibility criteria

We selected participants who (1) had been prescribed AOM between May 1, 2015, and December 31, 2022; (2) were diagnosed with bipolar disorder according to the International Classification of Diseases, 10th Revision (ICD‐10)^25^; (3) received AOM every consecutive month for more than 1 year; (4) had no missing medical data from both the pre‐ and post‐treatment periods; and (5) did not opt out of participating in the study. We excluded participants (1) diagnosed with schizophrenia, schizoaffective disorder, or delusional disorder according to the ICD‐10 and (2) whose administration interval of AOM was longer than 2 months.

### 2.4 Definitions of primary and secondary outcomes

The following four outcomes were established: rehospitalization rate, number of rehospitalizations, total hospitalization days, and time to rehospitalization. The proportion of patients readmitted at a participating psychiatric hospital at least once during the pre‐ or post‐treatment period was defined as the rehospitalization rate. The total number of psychiatric readmissions during each period was defined as the number of rehospitalizations. The total length of stay in the psychiatric hospital during each period was defined as the total hospitalization days. Finally, the time from the beginning of either the pre‐ or post‐treatment period to the first readmission was defined as the time to rehospitalization.

Primary outcomes included the rehospitalization rate, number of rehospitalizations, total hospitalization days, and time to rehospitalization in the context of overall psychiatric readmissions. In contrast, the secondary outcomes specifically examined these same measures in the context of manic or depressive readmissions and voluntary or involuntary rehospitalizations. The aim of the secondary outcomes was to investigate whether the effect of AOM on rehospitalization differed between manic and depressive admissions or between voluntary and involuntary admissions. Trained psychiatrists at each participating hospital collected data from medical records regarding mood episodes and hospitalization type at the time of readmission.

### 2.5 Definitions of the participant characteristics

Sociodemographic, clinical, and therapeutic data were collected. Sociodemographic variables were age, sex, education, and employment status at the time of AOM introduction. Clinical variables included age at the onset of bipolar disorder, duration of illness, and number of psychiatric hospitalizations from the onset of bipolar disorder to AOM introduction. Therapeutic variables were the annual prescription days of aripiprazole oral formulation during the year before the first AOM administration; maintenance dose of AOM after continuation of its administration for a year; and concurrent use of oral antipsychotics, mood stabilizers, and antidepressants after a year of AOM administration.

### 2.6 Consideration of biases

A common case‐reporting form was used at the participating hospitals to ensure that the information was collected comprehensively as per the uniform definitions in the research protocol. An individual double‐checked the data collected at each participating hospital.

### 2.7 Statistical methods

We used the R version 4.1.0. software (R Foundation for Statistical Computing) for statistical analyses. In descriptive statistics, normally distributed continuous variables are expressed as means and standard deviations, whereas non‐normally distributed data are expressed as medians and interquartile ranges. Meanwhile, categorical variables are expressed as numbers and percentages.

The following statistical analyses were conducted on the primary and secondary outcomes. McNemar's test was performed on the difference in the rehospitalization rates between the pre‐ and post‐treatment periods, and its effect size was evaluated using Cohen's g. The number of rehospitalizations and total hospitalization days were determined using the Shapiro–Wilk test for non‐normality. The differences between each period were examined using the Wilcoxon signed‐rank test, and non‐parametric effect sizes were evaluated using Cliff's delta. Cohen's g was assessed as follows: <0.05, ≥0.05 and <0.15, ≥ 0.15 and <0.25, and ≥0.25 corresponded to negligible, small, medium, and large, respectively.^26^ Moreover, Cliff's delta was considered small for values between 0.147 and 0.330, medium for values between 0.330 and 0.474, and large for values >0.474.^27^ To compare the time to rehospitalization between the pre‐ and post‐treatment periods, Kaplan–Meier survival plots were drawn for each period and tested with a log‐rank test. The two‐sided significance level for all tests was set at <0.05. For point estimates, 95% confidence intervals (CIs) were also provided. Furthermore, we conducted the same analysis on the subgroup of AOM users who were not concurrently receiving any concomitant antipsychotics, mood stabilizers, or antidepressants. This analysis aimed to evaluate the treatment effects of AOM by excluding the potential additional effects of concomitant medications.

### 2.8 Explanatory analysis

We explored the factors associated with rehospitalization after AOM by comparing groups with and without rehospitalization after AOM introduction. We tested for differences between the two groups with respect to sociodemographic, clinical, and therapeutic variables. The Fisher's exact test was used to compare categorical variables, and the Mann–Whitney *U* test was used to compare continuous variables. The significance level was set at a *p‐*value of <0.05.

## 3 RESULTS

### 3.1 Participant selection

**FIGURE 2:**
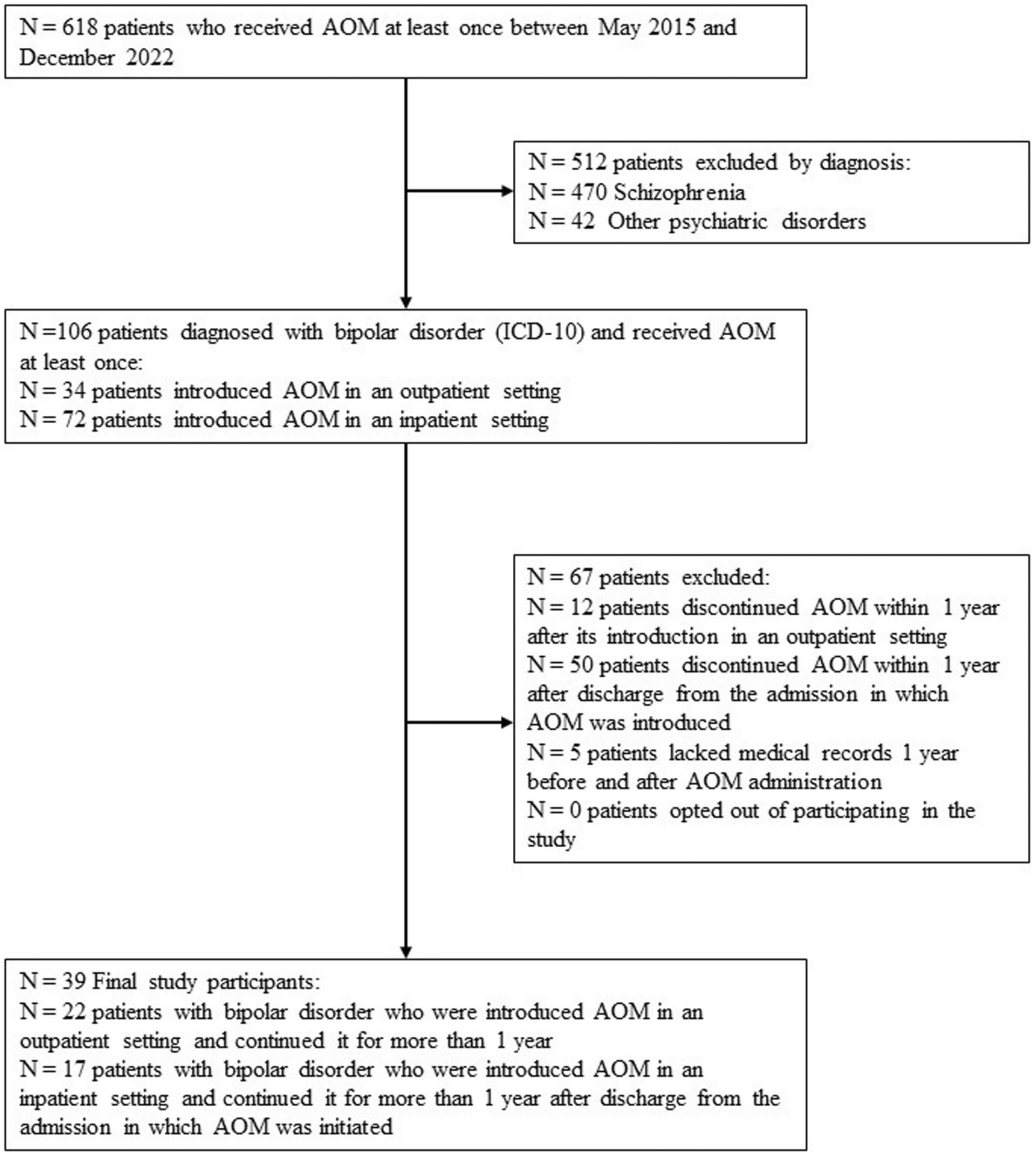
Selection process for study participants. ICD‐10, International Classification of Diseases, 10th Revision.

In total, 618 patients from the participating hospitals underwent AOM treatment at least once. Among them, 512 had non‐bipolar disorders, including schizophrenia. A total of 106 patients had bipolar disorder and had used AOM at least once, of whom 34 and 72 received AOM in outpatient and inpatient settings, respectively. Sixty‐seven patients were excluded because they had been receiving AOM for less than 1 year (*n* = 62) or had missing medical data (*n* = 5). Thirty‐nine patients were included in the final analysis (Figure 2).

### 3.2 Participant characteristics

The participant characteristics are summarized in Table 1. The average age of the study participants was 39.2 ± 15.6 years, and 30.8% were men. The mean age of onset of bipolar disorder was 28.5 ± 11.3 years, the mean duration of illness was 10.7 ± 8.5 years, and the median number of lifetime psychiatric hospitalizations before initiating AOM was 3.0, with an interquartile range of 1.0 to 4.5. The mean annual prescription days of aripiprazole oral formulation during the year before the first AOM administration was 172.7 ± 143.0 days. The mean maintenance dose of AOM after a year of its administration was 345.1 mg ± 74.4 mg. The number of patients who concurrently used at least one oral antipsychotic, mood stabilizer, or antidepressants after a year of AOM administration was 7/39 (17.9%), 15/39 (38.5%), and 6/39 (15.4%), respectively.

**TABLE 1:** Participant characteristics.

Variables	Number of participants *N* = 39
Age at AOM introduction, mean ± SD, years	39.2 ± 15.6
Male sex, *n* (%)	12 (30.8)
Education, mean ± SD, years	13.0 ± 1.9
Employed, *n* (%)	14 (35.9)
Age of onset of illness, mean ± SD, years	28.5 ± 11.3
Duration of illness, mean ± SD, years	10.7 ± 8.5
Number of lifetime psychiatric hospitalization, median [IQR]	3.0 [1.0–4.5]
Annual prescription days of oral aripiprazole, mean ± SD, days	172.7 ± 143.0
AOM maintenance dose, mean ± SD, mg	345.1 ± 74.4
Use of at least one concurrent oral antipsychotic, *n* (%)	7 (17.9)
Use of at least one concurrent mood stabilizer, *n* (%)	15 (38.5)
Lithium, *n* (%)	6 (15.4)
Valproate, *n* (%)	6 (15.4)
Lamotrigine, *n* (%)	5 (12.8)
Carbamazepine, *n* (%)	1 (2.6)
Use of at least one concurrent antidepressant, *n* (%)	6 (15.4)

*Note*: The number of lifetime psychiatric hospitalizations was counted from the onset of bipolar disorder to the first AOM administration. The annual prescription days of oral aripiprazole were recorded during the year before the first AOM administration. The AOM maintenance dose and concurrent use of oral antipsychotics, mood stabilizers, and antidepressants were assessed a year after AOM administration.

Abbreviations: AOM, aripiprazole once monthly; IQR, interquartile range; SD, standard deviation.

### 3.3 Outcome results

During the pre‐treatment period, 33 psychiatric rehospitalizations were recorded: 24/33 (73%) were attributed to mania, 9/33 (27%) to depression, 9/33 (27%) to voluntary admissions, and 24/33 (73%) to involuntary admissions. In contrast, the post‐treatment period saw 16 psychiatric rehospitalizations, with 4/16 (25%) related to mania and 12/16 (75%) to depression. Among these rehospitalizations, 12/16 (75%) were voluntary admissions, while 4/16 (25%) were classified as involuntary.

### 3.4 Results of the primary analysis

**FIGURE 3:**
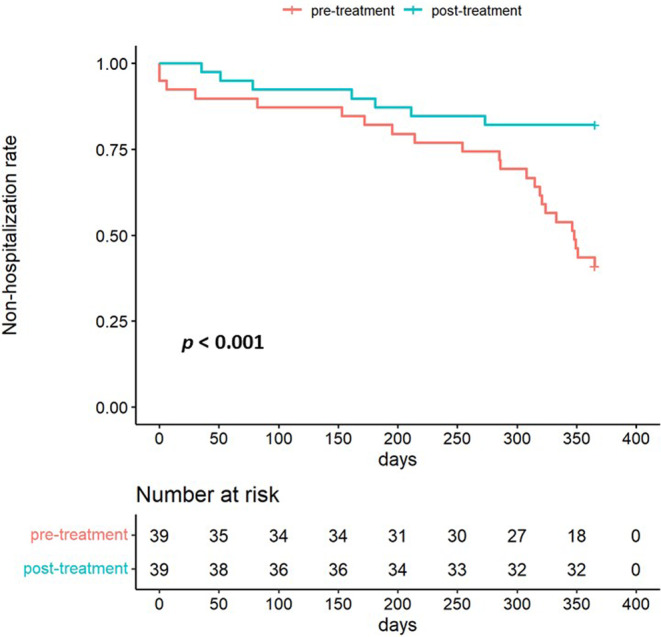
Time to rehospitalization before and after aripiprazole once monthly. Kaplan–Meier survival plots are shown comparing times to rehospitalization between the pre‐ and post‐treatment periods. The red survival plot represents the pre‐treatment period. The green survival plot represents the post‐treatment period. The log‐rank test was used to identify statistical significance (*p* < 0.001).

Table 2 presents the detailed results of the primary analysis focused on overall psychiatric readmissions. Rehospitalization rates decreased significantly from 23/39 (59%) to 7/39 (18%) (*p* = 0.001) between the pre‐ and post‐treatment periods, respectively, with a Cohen's g of 0.33 (95% CI: 0.14–0.43) for a large effect size. The number of rehospitalizations decreased significantly from a mean of 0.85 per person‐year to 0.41 per person‐year (*p* = 0.048) between the pre‐ and post‐treatment periods, with a medium effect size of 0.38 (95% CI: 0.16–0.56) for Cliff's delta. The total hospitalization days fell significantly from a mean of 34.9 days to 14.4 days (*p* = 0.008) between the pre‐ and post‐treatment periods, with a medium effect size of 0.38 (95% CI: 0.15–0.57) for Cliff's delta. Figure 3 compares the times to rehospitalization before and after AOM administration using Kaplan–Meier survival plots, and AOM significantly prolonged the time to rehospitalization (*p* < 0.001).

**TABLE 2:** Rehospitalization rate, number of rehospitalizations, and total hospitalization days related to overall psychiatric readmissions before and after aripiprazole once monthly in patients with bipolar disorder.

	Pre‐treatment	Post‐treatment	*p* value	Effect size (95% CI)
Rehospitalization rate	23/39 (59%)	7/39 (18%)	**0.001** ^a^	0.33 (0.14–0.43)^c^
Number of rehospitalizations				
Mean ± SD	0.85 ± 0.90	0.41 ± 1.14	**0.048** ^b^	0.38 (0.16–0.56)^d^
Median [IQR]	1.0[0.0–1.0]	0.0[0.0–0.0]		
Total hospitalization days				
Mean ± SD	34.9 ± 42.7	14.4 ± 36.5	**0.008** ^b^	0.38 (0.15–0.57)^d^
Median [IQR]	19[0.0–57.5]	0.0[0.0–0.0]		

*Note*: Bold values denote statistical significance at the level of *p* < 0.05. Effect sizes are expressed with point estimates and 95% CIs.

Abbreviations: CI, confidence interval; IQR, interquartile range; SD, standard deviation.

^a^ McNemar's test.

^b^ Wilcoxon signed rank test.

^c^ Cohen's g.

^d^ Cliff's delta.

### 3.5 Results of the secondary analysis

Rehospitalizations for mania exhibited significant decreases across all measures between the pre‐ and post‐treatment periods, while rehospitalizations for depression did not show any significant changes (Tables S1 and S2). Furthermore, AOM administration resulted in a notable improvement in the time to rehospitalization for mania (*p* < 0.001), whereas no significant improvement was observed for depression (*p* = 0.53) after AOM administration. Involuntary rehospitalizations demonstrated significant reductions across all measures during the pre‐ and post‐treatment periods, whereas no significant changes were observed for voluntary rehospitalizations (Tables S3 and S4). Additionally, AOM significantly prolonged the time to involuntary rehospitalization (*p* < 0.001), but no significant effect was noted for voluntary rehospitalizations (*p* = 0.76) in the pre‐ and post‐treatment period comparisons.

### 3.6 Results of the subgroup analysis

Twenty of 39 (51%) participants were AOM users not concurrently taking any antipsychotic, mood stabilizer, or antidepressant medications. This subgroup showed a significant reduction in rehospitalization across all measures between the pre‐ and post‐treatment periods (Table S5), along with a significant improvement in the time to rehospitalization (*p* < 0.001).

### 3.7 Results of the explanatory analysis

Seven and 32 patients were in the rehospitalization and non‐rehospitalization groups, respectively, after introducing AOM. Six of seven (86%) patients in the rehospitalization group and nine of 32 (28%) in the non‐rehospitalization group were on mood stabilizers after 1 year of AOM, with a significant difference between the two groups (*p* = 0.02). No significant differences were detected between the two groups for any other variables. Details are shown in Table S6.

## 4 DISCUSSION

### 4.1 Summary of the findings

This study found that AOM significantly reduced rehospitalization rates, number of rehospitalizations, and total hospitalization days and significantly prolonged the time to rehospitalization for patients with bipolar disorder in a 1‐year retrospective mirror‐image study. These findings remained consistent even in subgroup analyses that excluded concomitant medications with maintenance effects. Using the same 1‐year mirror image design, previous studies have shown that risperidone LAI, paliperidone LAI, and overall SGA‐LAIs significantly reduced rehospitalization for bipolar disorder.^18^, ^19^, ^20^, ^21^, ^22^ However, these previous studies did not specifically reveal the preventive effect of AOM on rehospitalization. To the best of our knowledge, this study is the first to demonstrate the preventive effect of AOM on rehospitalization for bipolar disorder in the real‐world setting. Additionally, this study revealed that AOM reduced rehospitalizations due to mania and involuntary readmission. Therefore, AOM holds clinical value as a maintenance treatment for bipolar disorder in preventing psychiatric rehospitalization, especially in the context of manic episodes and involuntary admissions.

### 4.2 Interpretation of the primary analysis

The effect of AOM on rehospitalization for bipolar disorder shown in this study is consistent with the results of several previous studies. In the present study, the rehospitalization rate significantly decreased from 59% to 18% between the pre‐ and post‐treatment periods with AOM introduction; likewise, the rehospitalization rate significantly decreased from 57% to 23% in the SGA‐LAI subgroup of the STAR study (*n* = 52).^20^ The number of rehospitalizations significantly decreased from 0.85 to 0.41 in the present study and from 0.9 to 0.1 in the Turkish study (*n* = 17).^21^ The more significant reduction in the Turkish study may have been caused by differences in the participants' background characteristics between the two studies. The total hospitalization days were reduced from 34 to 14 days in the present study and from 30 to 17 days in the SGA‐LAI subgroup of the NOMIAC study (*n* = 49)^22^; thus, both studies have similar findings.

The STAR^20^ and NOMIAC studies,^22^ conducted in Italy, did not reveal an AOM‐specific reduction in rehospitalization for bipolar disorder, which is not congruent with the findings of the present study. Both studies included a relatively large number of AOM users: 30/71 (42.3%) in the STAR study and 28/68 (54.9%) in the NOMIAC study. Moreover, both studies included 20–25% of non‐adherent patients who received LAIs for less than 12 months, and there was no significant reduction in rehospitalization in the LAI non‐adherent subgroup. These studies may have underestimated the AOM‐specific reduction in rehospitalization owing to the inclusion of non‐adherent AOM users. However, since the present study excluded non‐adherent patients, we could determine the preventive effect of AOM on rehospitalization post‐treatment.

In the present study, the time to rehospitalization was significantly prolonged between the pre‐ and post‐treatment periods. The survival curve for rehospitalization in the pre‐treatment period showed a cumulative decrease after 300 days, whereas that in the post‐treatment period remained flat beyond 300 days. Medication non‐adherence is a potential reason for the increase in rehospitalization after 300 days in the pre‐treatment period. In a study examining oral medication adherence among patients with bipolar disorder using Japanese health insurance data, the 1‐year average PDC ranged from 0.51 to 0.61, which is below 0.80, the PDC that indicates good adherence.^28^ Thus, non‐adherence with oral medication during the pre‐treatment period may result in insufficient therapeutic concentrations, leading to recurrence and rehospitalization.^12^ Conversely, AOM prevented recurrence and rehospitalization in the post‐treatment period because the formulation maintained sufficient therapeutic concentrations for 1 month.^29^

### 4.3 Interpretation of the secondary analysis

The findings of this study demonstrate a significant reduction in manic rehospitalizations in real‐world settings with the use of AOM. However, statistical significance was not achieved for depressed rehospitalizations. These results are consistent with those of previous studies. For instance, a 52‐week double‐blind randomized controlled trial showed that AOM significantly reduced the recurrence of manic and mixed episodes but did not significantly reduce the recurrence of depressive episodes.^14^ Similarly, a 100‐week double‐blind randomized controlled trial of oral aripiprazole revealed a significant reduction in manic episodes but not in depressive episodes.^30^ Correspondingly, the 1‐year retrospective mirror image study reported a significant decrease in hospitalization days for mania before and after the introduction of LAIs, including AOM. However, no significant difference was observed in the number of hospitalization days for depression.^22^ Based on these collective findings, it appears AOM is effective in preventing manic episodes but may not be as effective in preventing depressive episodes.

### 4.4 Interpretation of the explanatory analysis

The rehospitalization group following the introduction of AOM exhibited a significantly higher likelihood of utilizing concomitant mood stabilizers than the non‐rehospitalization group. The majority (75%) of rehospitalizations after AOM were attributed to depression, indicating the concurrent use of mood stabilizers served as a treatment approach for recurrence/relapse of depression following AOM introduction. In cases where the initiation of mood stabilizers was delayed, the worsening of depression may have led to rehospitalization. A network meta‐analysis suggested that the combination of oral aripiprazole with either valproate or lamotrigine was more effective in preventing recurrence/relapse of the depressed phase in bipolar disorder compared to AOM alone.^31^ Therefore, early implementation of these mood stabilizers in combination with AOM introduction may help reduce rehospitalization for depression in patients with bipolar disorder experiencing a predominantly depressed phase.^32^

### 4.5 Limitations

The findings of this study should be interpreted with caution owing to its limitations. First, although the number of AOM users in this study was larger than that in previous studies, it was still lower at 39. Second, because the study participants were recruited from six psychiatric emergency and acute care hospitals, the results of this study are applicable to severely ill patients with bipolar disorder and may not be generalizable. Third, as this study did not include patients with bipolar disorder who discontinued AOM within 1 year, it may have excluded patients who had an unfavorable response to AOM within 1 year, leading to selection bias and overestimation of the treatment effect. Furthermore, although we diagnosed bipolar disorder based on medical record data, we lacked sufficient information to distinguish bipolar disorder types I and II. We determined the mood episodes at readmission (manic or depressive); however, we were unable to assess the severity of these episodes using established rating scales such as the Young Mania Rating Scale or Hamilton Depression Scale. Additionally, our study did not include the observation of recurrence/relapse events not requiring rehospitalization. We were also unable to adjust for risk factors for rehospitalization, such as substance abuse and personality disorders,^33^, ^34^ and protective factors for rehospitalizations, such as psychoeducation.^35^, ^36^

## 5 CONCLUSION

Our study found that AOM significantly reduced rehospitalization rates, number of rehospitalizations, and total hospitalization days and significantly prolonged the time to rehospitalization in the context of overall psychiatric readmissions among patients with bipolar disorder by comparing data from 1 year before and after AOM treatment. These findings suggest that AOM may help prevent rehospitalization as a maintenance treatment for bipolar disorder. Future studies should examine the robustness and persistence of the rehospitalization preventive effect of AOM with larger sample sizes and extended observation periods beyond 1 year.

## AUTHOR CONTRIBUTIONS

Goto J. and Nabeshima Y. conceptualized and designed the study. Goto J., Shono M., Abe Y., Fujita Y., Ueda K., and Yoshida B. were involved in the data acquisition. Goto J., Shono M., and Nabeshima Y. interpreted data for the work. Goto J. prepared the manuscript. Shono M., Abe Y., Fujita Y., Ueda K., Yoshida B., and Nabeshima Y. critically revised the manuscript for important intellectual content. All authors approved the version to be published and agreed to be accountable for all aspects of this work.

## FUNDING INFORMATION

This study did not receive any specific grants from funding agencies in the public, commercial, or non‐profit sectors.

## CONFLICT OF INTEREST STATEMENT

Goto J. received lecture fees from Otsuka Pharmaceutical Co., Ltd., Janssen Pharmaceutical K.K., Sumitomo Pharma Co., Ltd., and Meiji Seika Pharma Co., Ltd.; Shono M. received lecture fees from Otsuka Pharmaceutical Co., Ltd., Sumitomo Pharma Co., Ltd., and Nippon Boehringer Ingelheim Co., Ltd.; Abe Y. received lecture fees from Otsuka Pharmaceutical Co., Ltd., Janssen Pharmaceutical K.K., Sumitomo Pharma Co., Ltd., MSD K.K., Viatris Inc., Meiji Seika Pharma Co., Ltd., Takeda Pharmaceutical Co., Ltd., Yoshitomiyakuhin Corporation, and EA Pharma Co., Ltd.; Fujita Y. received lecture fees from Otsuka Pharmaceutical Co., Ltd., Sumitomo Pharma Co., Ltd., Janssen Pharmaceutical K.K., Meiji Seika Pharma Co., Ltd., Takeda Pharmaceutical Co., Ltd., Viatris Inc., Eisai Co., Ltd., and MSD K.K.; Ueda K. declared no conflicts of interest; Yoshida B. received a lecture fee from Otsuka Pharmaceutical Co., Ltd.; and Nabeshima Y. received lecture fees from Otsuka Pharmaceutical Co., Ltd., Eisai Co., Ltd., Takeda Pharmaceutical Co., Ltd., Sumitomo Pharma Co., Ltd., MSD K.K., Meiji Seika Pharma Co., Ltd., Janssen Pharmaceutical K.K., and Yoshitomiyakuhin Corporation and an advisory fee from Sumitomo Pharma Co., Ltd.

## ETHICS STATEMENT

Approval of the research protocol by an Institutional Reviewer Board: The protocol for this research project has been approved by the suitably constituted Ethics Committees of Yuge Hospital (Approval No. 233), and it conforms to the provisions of the Declaration of Helsinki.

Informed consent: The opt‐out method of consent was adopted, wherein the study information was posted on institutional websites as a clinical study and patients were provided with an adequate period to opt out.

Registry and the registration no. of the study/trial: N/A.

Animal Studies: N/A.

## Supporting information


Tables S1‐S6.

## Data Availability

The data of the present study cannot be made publicly available because the disclosure of personal data was not included in the research protocol and because the opt‐out clearly states that personal data will not be disclosed.
