# Supplementary material for: Preventive effect of aripiprazole once monthly on rehospitalization for bipolar disorder: A multicenter 1‐year retrospective mirror image study
Source: Neuropsychopharmacol Rep. 2023 Aug 10;43(3):425–33. doi: 10.1002/npr2.12371 (PMC10496053; doi:10.1002/npr2.12371)
Supplement: Supplementary file 1 — Tables S1‐S6. [file NPR2-43-425-s001.docx]

Supplemental Table 1. Rehospitalization rate, number of rehospitalizations, and total hospitalization days related to mania before and after aripiprazole once monthly in patients with bipolar disorder

|  | Pre-treatment | Post-treatment | *p* value | Effect size (95% CI) |
| --- | --- | --- | --- | --- |
| Rehospitalization rate  related to mania | 18/39 (46%) | 4/39 (10%) | **< 0. 001**^†^ | 0.39 (0.17-0.47)^§^ |
| Number of rehospitalizations  related to mania  mean±SD  median [IQR] | 0.62±0.75  0.0[0.0-1.0] | 0.10±0.31  0.0[0.0-0.0] | **< 0. 001**^‡^ | 0.37 (0.18-0.54)^¶^ |
| Total hospitalization days  related to mania  mean±SD  median [IQR] | 23.9±35.1  0.0[0.0-43.0] | 6.26±22.6  0.0[0.0-0.0] | **0. 02**^‡^ | 0.35 (0.15-0.53)^¶^ |

Notes: Bold and underlined values denote statistical significance at the level of *p* < 0.05.

Effect sizes are expressed with point estimates and 95% CIs.

^†^McNemar’s test, ^‡^Wilcoxon signed rank test, ^§^Cohen’s g, ^¶^Cliff’s delta

Abbreviations: SD = standard deviation; IQR = interquartile range; CI = confidence interval

Supplemental Table 2. Rehospitalization rate, number of rehospitalizations, and total hospitalization days related to depression before and after aripiprazole once monthly in patients with bipolar disorder

|  | Pre-treatment | Post-treatment | *p* value | Effect size (95% CI) |
| --- | --- | --- | --- | --- |
| Rehospitalization rate  related to depression | 7/39 (18%) | 5/39 (13%) | 0.53^†^ | 0.10 (-0.19-0.33)^§^ |
| Number of rehospitalizations  related to depression  mean±SD  median [IQR] | 0.23±0.54  0.0[0.0-0.0] | 0.31±1.1  0.0[0.0-0.0] | 0. 75^‡^ | 0.043 (-0.11-0.20)^¶^ |
| Total hospitalization days  related to depression  mean±SD  median [IQR] | 11.0±27.7  0.0[0.0-0.0] | 8.13±24.2  0.0[0.0-0.0] | 0. 85^‡^ | 0.047 (-0.12-0.21)^¶^ |

Notes: Bold and underlined values denote statistical significance at the level of *p* < 0.05.

Effect sizes are expressed with point estimates and 95% CIs.

^†^McNemar’s test, ^‡^Wilcoxon signed rank test, ^§^Cohen’s g, ^¶^Cliff’s delta

Abbreviations: SD = standard deviation; IQR = interquartile range; CI = confidence interval

Supplemental Table 3. Rehospitalization rate, number of rehospitalizations, and total hospitalization days related to involuntary admission before and after aripiprazole once monthly in patients with bipolar disorder

|  | Pre-treatment | Post-treatment | *p* value | Effect size (95% CI) |
| --- | --- | --- | --- | --- |
| Rehospitalization rate  related to involuntary admission | 20/39 (51%) | 3/39 (8%) | **< 0. 001**^†^ | 0.45 (0.25-0.49)^§^ |
| Number of rehospitalizations  related to involuntary admission  mean±SD  median [IQR] | 0.62±0.67  1.0[0.0-1.0] | 0.10±0.38  0.0[0.0-0.0] | **< 0. 001**^‡^ | 0.43 (0.23-0.60)^¶^ |
| Total hospitalization days  related to involuntary admission  mean±SD  median [IQR] | 27.4±35.5  10[0.0-52] | 7.87±27.7  0.0[0.0-0.0] | **0. 006**^‡^ | 0.40 (0.20-0.58)^¶^ |

Notes: Bold and underlined values denote statistical significance at the level of *p* < 0.05.

Effect sizes are expressed with point estimates and 95% CIs.

^†^McNemar’s test, ^‡^Wilcoxon signed rank test, ^§^Cohen’s g, ^¶^Cliff’s delta

Abbreviations: SD = standard deviation; IQR = interquartile range; CI = confidence interval

Supplemental Table 4. Rehospitalization rate, number of rehospitalizations, and total hospitalization days related to voluntary admission before and after aripiprazole once monthly in patients with bipolar disorder

|  | Pre-treatment | Post-treatment | *p* value | Effect size (95% CI) |
| --- | --- | --- | --- | --- |
| Rehospitalization rate  related to voluntary admission | 5/39 (13%) | 6/39 (15%) | 0.74^†^ | 0.06 (-0.23-0.31)^§^ |
| Number of rehospitalizations  related to voluntary admission  mean±SD  median [IQR] | 0.23±0.74  0.0[0.0-0.0] | 0.31±1.0  0.0[0.0-0.0] | 0. 62^‡^ | -0.02 (-0.17-0.12)^¶^ |
| Total hospitalization days  related to voluntary admission  mean±SD  median [IQR] | 7.51±26.5  0.0[0.0-0.0] | 6.51±19.6  0.0[0.0-0.0] | 0. 70^‡^ | -0.02 (-0.18-0.13)^¶^ |

Notes: Bold and underlined values denote statistical significance at the level of *p* < 0.05.

Effect sizes are expressed with point estimates and 95% CIs.

^†^McNemar’s test, ^‡^Wilcoxon signed rank test, ^§^Cohen’s g, ^¶^Cliff’s delta

Abbreviations: SD = standard deviation; IQR = interquartile range; CI = confidence interval

Supplemental Table 5. Rehospitalization rate, number of rehospitalizations, and total hospitalization days for the subgroup of patients with bipolar disorder using aripiprazole once monthly without concomitant medications

|  | Pre-treatment | Post-treatment | *p* value | Effect size (95% CI) |
| --- | --- | --- | --- | --- |
| Rehospitalization rate  of the subgroup | 11/20 (55%) | 1/20 (5%) | **0.004**^†^ | 0.42 (0.15-0.49)^§^ |
| Number of rehospitalizations  of the subgroup  mean±SD  median [IQR] | 0.70±0.73  1.0[0.0-1.0] | 0.05±0.22  0.0[0.0-0.0] | **0.005**^‡^ | 0.51 (0.23-0.71)^¶^ |
| Total hospitalization days  of the subgroup  mean±SD  median [IQR] | 26.2±30.3  11.5[0.0-51.5] | 0.950±4.25  0.0[0.0-0.0] | **0.001**^‡^ | 0.52 (0.24-0.72)^¶^ |

Notes: Bold and underlined values denote statistical significance at the level of *p* < 0.05.

Effect sizes are expressed with point estimates and 95% CIs.

^†^McNemar’s test, ^‡^Wilcoxon signed rank test, ^§^Cohen’s g, ^¶^Cliff’s delta

Abbreviations: SD = standard deviation; IQR = interquartile range; CI = confidence interval

Supplemental Table 6. Factors associated with rehospitalization for bipolar disorder in the post-treatment period

| Variables | Rehospitalization group in the post-treatment period  N = 7 | Non-rehospitalization group in the post-treatment period  N = 32 | *p* value |
| --- | --- | --- | --- |
| Age at AOM introduction, mean ± SD, years | 36.9 ± 15.8 | 39.7 ± 15.8 | 0.67^‡^ |
| Male sex, n (%) | 2 (28.6) | 10 (31.2) | 1.00^†^ |
| Education, mean ± SD, years | 13.1 ± 2.5 | 13.0 ± 1.8 | 0.86^‡^ |
| Employed, n (%) | 4 (57.1) | 10 (31.2) | 0.39^†^ |
| Age at onset of illness, mean ± SD, years | 23.6 ± 5.1 | 29.5 ± 12.0 | 0.21^‡^ |
| Duration of illness, mean ± SD, years | 13.3 ± 11.8 | 10.1 ± 7.8 | 0.38^‡^ |
| Lifetime psychiatric hospitalizations, median [IQR] | 3.0 [2.0-4.0] | 2.5 [1.0-4.3] | 0.32^‡^ |
| Annual prescription days of oral aripiprazole, mean ± SD | 183.1 ± 132.7 | 170.4 ± 147.1 | 0.83^‡^ |
| AOM maintenance dose, mean ± SD, mg | 385.7 ± 37.8 | 336.2 ± 77.8 | 0.11^‡^ |
| Use of at least one concurrent oral antipsychotic, n (%) | 1 (14.3) | 6 (18.8) | 1.00^†^ |
| Use of at least one concurrent mood stabilizer, n (%) | 6 (85.7) | 9 (28.1) | **0.02**^†^ |
| Use of at least one concurrent antidepressant, n (%) | 0 (0.0) | 6 (18.8) | 0.50^†^ |

Notes: Lifetime psychiatric hospitalizations were counted from the onset of bipolar disorder to the first AOM administration. The annual prescription days of oral aripiprazole were recorded during the year before the first AOM administration. The AOM maintenance dose and concurrent use of oral antipsychotics, mood stabilizers, and antidepressants were assessed a year after AOM administration.

Bold and underlined values denote statistical significance at the level of *p* < 0.05. ^†^Fisher’s exact test, ^‡^Mann–Whitney U test

Abbreviations: SD = standard deviation; IQR = interquartile range; AOM = aripiprazole once monthly
